# The lncRNA XIST/miR‐125b‐2‐3p axis modulates cell proliferation and chemotherapeutic sensitivity via targeting Wee1 in colorectal cancer

**DOI:** 10.1002/cam4.3777

**Published:** 2021-03-05

**Authors:** Zhao‐lei Zeng, Jia‐huan Lu, Yun Wang, Hui Sheng, Ying‐nan Wang, Zhan‐hong Chen, Qi‐nian Wu, Jia‐Bo Zheng, Yan‐xing Chen, Dong‐dong Yang, Kai Yu, Hai‐yu Mo, Jia‐jia Hu, Pei‐shan Hu, Ze‐xian Liu, Huai‐qiang Ju, Rui‐Hua Xu

**Affiliations:** ^1^ State Key Laboratory of Oncology in South China Collaborative Innovation Center for Cancer Medicine Sun Yat‐sen University Cancer Center Guangzhou China; ^2^ Department of Medical Oncology Sun Yat‐sen University Cancer Center Guangzhou China; ^3^ Department of Medical Oncology and Guangdong Key Laboratory of Liver Disease the Third Affiliated Hospital of Sun Yat‐sen University Guangzhou China; ^4^ Department of Pathology Sun Yat‐sen University Cancer Center Guangzhou China

**Keywords:** chemotherapeutic sensitivity, colorectal cancer, drug resistance, miR‐125b‐2‐3p

## Abstract

**Background:**

Numerous reports on microRNAs have illustrated their role in tumor growth and metastasis. Recently, a new prognostic factor, miR‐125b‐2‐3p, has been identified for predicting chemotherapeutic sensitivity in advanced colorectal cancer (CRC). However, the specific mechanisms and biological functions of miR‐125b‐2‐3p in advanced CRC under chemotherapy have yet to be elucidated.

**Methods:**

MiR‐125b‐2‐3p expression was detected by real‐time PCR (RT‐PCR) in CRC tissues. The effects of miR‐125b‐2‐3p on the growth, metastasis, and drug sensitivity of CRC cells were tested in vitro and in vivo. Based on multiple databases, the upstream competitive endogenous RNAs (ceRNAs) and the downstream genes for miR‐125b‐2‐3p were predicted by bioinformatic analysis, followed by the experiments including luciferase reporter assays, western blot assays, and so on.

**Results:**

MiR‐125b‐2‐3p was significantly lowly expressed in the tissues and cell lines of CRC. Higher expression of miR‐125b‐2‐3p was associated with relatively lower proliferation rates and fewer metastases. Moreover, overexpressed miR‐125b‐2‐3p remarkably improved chemotherapeutic sensitivity of CRC in vivo and in vitro. Mechanistically, miR‐125b‐2‐3p was absorbed by long noncoding RNA (lncRNA) XIST regulating WEE1 G2 checkpoint kinase (WEE1) expression. The upregulation of miR‐125b‐2‐3p inhibited the proliferation and epithelial‐mesenchymal transition (EMT) of CRC induced by lncRNA XIST.

**Conclusions:**

Lower miR‐125b‐2‐3p expression resulted in lower sensitivity of CRC to chemotherapy and was correlated with poorer survival of CRC patients. LncRNA XIST promoted CRC metastasis acting as a ceRNA for miR‐125b‐2‐3p to mediate WEE1 expression. LncRNA XIST‐miR‐125b‐2‐3p‐WEE1 axis not only regulated CRC growth and metastasis but also contributed to chemotherapeutic resistance to CRC.

## Graphical abstract

Low expression of miR‐125b‐2‐3p in CRC was linked to lower chemotherapeutic sensitivity and poor survival. The lncRNA XIST promoted CRC invasion and migration by functioning as a ceRNA for miR‐125b‐2‐3p to mediate WEE1 expression. Our findings suggest that the lncRNA XIST‐miR‐125b‐2‐3p‐WEE1 axis not only regulated CRC growth and metastasis but also contributed to chemotherapeutic resistance to CRC, which may shed light on their targeted applications.

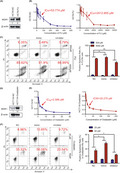

## 1 INTRODUCTION

Based on the cancer statistics in 2018, colorectal cancer (CRC) ranked third in terms of incidence and second in terms of mortality among all types of cancers worldwide.^1^ In China, CRC ranks fourth in incidence and mortality.^2^ Currently, chemotherapy and radiotherapy are chosen as the primary treatments for advanced CRC. However, the 5‐year survival rate of patients with advanced CRC is limited due to severe drug resistance low efficiency and a high rate of metastasis. Therefore, novel and useful biomarkers are urgently needed to predict the development of advanced CRC and explore the related drug‐resistance mechanisms.

Less than 3% of the human genome has been identified as protein‐coding gene exons, leaving 97% of the remaining territory to be explored. Intergenic sequences, once referred to as “junk DNA,” have revealed a close relationship with many diseases.^3^ However, many complex molecular mechanisms remain elusive, especially in tumor evolution, development, and drug resistance. In the GENCODE database, 7,053 small RNAs have been annotated, and 28% of them are expressed in at least one cell line.^4^ Abundant studies have confirmed that microRNAs are related to the development and drug resistance of CRC, but there are still few efficient indicators to guide clinical treatments. Moreover, a large number of novel long RNAs are expected to have dual roles: 1) protein‐coding RNAs; 2) precursors for many small RNAs. In recent years, a deeper understanding of noncoding RNAs (ncRNAs) has revealed that long noncoding RNAs (lncRNAs) can be used as competitive endogenous RNAs (ceRNAs; binding endogenous RNA) to absorb small RNAs, thereby influencing the expression of downstream target genes. Hence, elucidating the pathogenic and drug‐resistance mechanisms of advanced CRC through noncoding RNAs would improve individual treatments.

Based on historical reports, five circulating microRNAs, including miR‐125b‐2, are consistently altered in acute stroke, which might be useful and sensitive tools for the diagnosis of stroke.^5^ Moreover, a team confirmed that miR‐125b‐2 was upregulated in CRC with liver metastasis.^6^ In recent studies, hsa‐miR‐125b‐2‐3p was found to be linked with the pathological stage of CRC.^7^ Additionally, through an investigation on the mechanisms of miRNA profile in hematopoietic stem cells (HSCs), one team found that rno‐miR‐125b‐2‐3p was downregulated after tensile strain was applied to activated HSCs.^8^ In light of a previous study, we developed and verified a novel classifier based on two miRNAs, miR‐933 and miR‐125b‐2‐3p. They are capable of predicting disease progression and the benefit of receiving standard first‐line chemotherapy in advanced CRC. Additionally, based on the signature, patients categorized in the high‐risk group had a higher chance of disease progression and worse survival outcomes after chemotherapy.^9^ In this research, we further revealed that high levels of miR‐125b‐2‐3p could diminish the tumor's ability to proliferate and metastasize. Furthermore, the ncRNA XIST acted as a sponge RNA and impacted the function of miR‐125b‐2‐3p, thus regulating the WEE1 G2 checkpoint kinase (WEE1) targeted genes. Our results demonstrated that LncRNA XIST‐miR‐125b‐2‐3p‐WEE1 axis not only regulated CRC growth and metastasis but also contributed to chemotherapeutic resistance to CRC.

## 2 MATERIALS AND METHODS

### 2.1 Human tissue samples

One hundred twenty‐two tissues were obtained from patients who were newly diagnosed with CRC and received surgical resection at the Sun Yat‐sen University Cancer Center (SYSUCC) from Feb 2002 to Jan 2011. All samples were confirmed by pathologists. Informed consent was provided from all patients enrolled. This study was approved by the institutional ethics review board of SYSUCC (Guangzhou, China). Histological grade was staged in terms of the seventh TNM staging of the International Union against Cancer (UICC)/American Joint Committee on Cancer (AJCC) system.

### 2.2 Cell lines and cell culture

Human CRC cell lines, including HCT116, DLD1, and HCT8, were purchased from the American Type Culture Collection (Manassas, VA, USA). Based on the instructions provided: all cells were cultured in RPMI‐1640 medium (GIBCO, Grand Island, NE, USE) with 10% fetal bovine serum (FBS, Thermo Fisher, Carlsbad, California, USA) at 37°C, 5% CO_2_. 0.2% DMSO as control of treatments in all experiments. Two kinds of drug‐resistant CRC cell lines were used, including the HCT116 oxaliplatin‐resistant and HCT8 5‐FU‐resistant cell lines. The culture concentration of HCT116 in oxaliplatin was 30 μl/ml, and HCT8 in 5‐FU was 70 μl/ml. We tested the drug resistance by observing the fold changes in IC_50_ values.

### 2.3 RNA isolation and real‐time PCR analysis

Total RNA from tissues and cells was extracted with TRIzol reagent (Takara, Otsu, Japan) according to the manufacturer's instructions.^10^ The concentration and purity of RNA were measured using a NanoDrop ONE spectrophotometer (Thermo Fisher Scientific, Wilmington, DE, USA). The reverse transcription of WEE1 and lncRNA XIST was performed using the High‐Capacity cDNA Reverse Transcription Kit (Applied Biosystems, Foster City, CA, USA). The transcription of microRNA was performed by the All‐in‐One First‐Strand Synthesis Kit (GeneCopoeia, Rockville, Montgomery, USA). A total of 2 μg RNA from each sample was transcribed for PCR detection. Total RNA was transcribed by a reverse transcription kit purchased from Takara. Real‐time PCR was carried out with SYBR Green Real‐Time PCR Master Mix (Toyobo, Osaka, Japan) using GAPDH as the reference. Real‐time PCR for microRNA was carried out by the All‐in‐One miRNA qRT‐PCR Detection Kit (GeneCopoeia, Rockville, Montgomery, USA) with U48 as a reference for microRNA. Real‐time qPCR was performed with the Roche 480 qPCR System (Roche Diagnostics, Basel, Switzerland), and fold changes were calculated according to the relative quantification 2^−∆CT^ method.

### 2.4 Cell transfections

The experiments were performed according to a simple procedure. In brief, approximately 2 × 10^5^ cells were cultured in a well of a 6‐well plate on the first day. On the second day, cell transfections were performed using 50 nM Lipofectamine 2000 (Invitrogen, Carlsbad, CA, USA), and the cells were collected after 48 hours. The miR‐125b‐2‐3p mimic, miR‐125b‐2‐3p inhibitor, positive and negative control of microRNA, and lncRNA inhibitor were bought from GenePharma (Shanghai, China). In addition, a lentivirus to overexpress WEE1 was constructed by OBiO (Shanghai, China).

### 2.5 Cell proliferation assay

For each sample, 3 × 10^3^ Cells were seeded onto a well of a 96‐well plate. After the incubation for 24 hours, the cells were stained with 20 μl of sterile 3‐(4,5‐dimethylthiazol‐2‐yl)‐5‐(3‐carboxymethoxyphenyl)‐2‐(4‐sulfophenyl)‐2H‐tetrazolium (MTS) dye (Promega, Madison, WI, USA) at 37°C for 2 hours. Finally, the absorbance was detected at a wavelength of 490 nm.

### 2.6 Cell colony formation assay

The cells were trypsinized and suspended in RPMI‐1640 medium supplemented with 10% FBS. The cells were then seeded in 6‐well plates in triplicate and incubated for 14 days at 37°C. After being washed twice with phosphate‐buffered saline (PBS), the cells were fixed for 30 minutes with methanol and stained for 1 hour with 0.1% crystal violet (1 mg/ml). Only the colonies containing >50 cells were counted, and the mean colony numbers were calculated.

### 2.7 Wound healing assay

CRC cells were equally trypsinized and seeded in 6‐well plates. After 12 hours, several artificial wounds were created using a 200 μl pipette tip. The wound healing distance was measured by taking pictures under a microscope after 24, 48, and 72 hours of incubation in the medium without FBS. The rate of cell coverage was evaluated by the changes in a series of daily records.

### 2.8 Cell migration and invasion assays (Transwell assay)

CRC cells were first trypsinized and counted. 1 × 10^5^ cells were plated into the upper chamber in 100 μl of medium without serum, while the lower chamber was filled with 500 μl of medium supplemented with 20% FBS. After culturing for approximately 24 hours, a portion of the cells invading the lower chamber were fixed with methanol and dyed with 0.1% crystal violet. Finally, these cells were observed under an inverted microscope (magnification 200×) and counted by analyzing five random views.

### 2.9 Flow cytometry analysis

The cell apoptosis and cell cycle were measured by flow cytometry following the manufacturer's instructions. In brief, cells in 6‐well plates were treated with different concentrations of 5‐FU or oxaliplatin. Cell apoptosis was assayed by an Annexin V/PI kit and cell cycle distribution was assayed by a cell cycle assay kit (KeyGen, Nanjing, China), both of which were followed by flow cytometry analysis (Beckman Coulter, California, USA). The data were evaluated by ModFit and CytExpert.

### 2.10 Vector construction and lentiviral transduction

The human WEE1 gene was PCR‐amplified from gDNA and cloned into a pLV‐puro lentiviral vector, synthesized by OBiO Technology (Shanghai, China) Corp., Ltd. HCT116 and DLD1 cells were transduced with the lentivirus according to the manufacturer's instructions. Subsequently, these cells were selected with puromycin (2 μg/ml) for 2 weeks. The overexpressed efficiency was confirmed using qRT‐PCR and western blot analysis.

### 2.11 Luciferase reporter assay

DNA fragments from a portion of lncRNA XIST, lncRNA‐XIST‐mut1, lncRNA‐XIST‐mut2, and lncRNA‐XIST‐mut1&2 were cloned into different pGL3‐basic vectors purchased from OBiO (Shanghai, China). The same procedure was followed for WEE1, WEE1‐mut1, WEE1‐mut2, and WEE1‐mut1&2. Ten thousand cells were seeded in triplicate in 96‐well plates and incubated for 24 hours. Then, the reporter plasmids (100 mg) and 5 ng of the pRL‐TK Renilla plasmid (OBiO, Shanghai, China) were added. The miR‐125b‐2‐3p mimic or NC was cotransfected in CRC cells using the Lipofectamine LTX reagent (Invitrogen, Carlsbad, USA). Luciferase and Renilla signals were measured 24 hours after transfection with the Dual‐Luciferase Reporter Assay Kit (Promega, Madison, USA) according to the standard protocol.^11^

### 2.12 Western blot analyses

The cellular protein was extracted in a radioimmunoprecipitation assay (RIPA) and quantified using a BCA assay (Thermo Fisher Scientific, Carlsbad, USA). Equal amounts of samples were separated by SDS‐PAGE before being transferred to polyvinylidene fluoride membranes (Immobilon‐P, Millipore, Bedford, USA). The membranes were then blocked for 1 hour at room temperature with 5% nonfat milk in Tris‐buffered saline plus Tween‐20 (TBST) and incubated at 4°C overnight with the diluted primary antibody. After the membranes were washed with TBST and incubated for 1 hour at room temperature with the diluted secondary antibody. Finally, enhanced chemiluminescence (SuperSignal ECL, Thermo Fisher Scientific, Carlsbad, USA) was added to visualize proteins in the membrane.

### 2.13 MicroRNA FISH

Oligonucleotide probes complementary to hsa‐miR‐125b‐2‐3p were purchased from ExonBio Lab (Guangzhou, China). The tissue sections were deparaffinized, dehydrated, and immersed in 0.2 N HCl for 20 min. After the slides were immersed in 0.5% Tween (PBS) solution, they were fixed in 10% neutral‐buffered formalin. Fixed tissues were treated with 200 μg/ml of Proteinase K at 37°C for 5 minutes, but the culture samples on glass cover slides were only treated with 0.1% Triton X‐100/PBS. The slides were then immersed in RNase‐free water for 3 minutes and air‐dried, followed by prehybridization in hybridization buffer for 2 hours at 37°C and hybridization with the probe for 24 hours. Subsequently, the slides were washed three times in 2× SSC with 0.5% Tween‐20 and miRNA in situ hybridization (MISH) signals were detected with an enzyme‐labeled fluorescence (ELF) signal amplification kit (Invitrogen, San Diego, CA). The slides were counterstained with DAPI (ProLong^TM^ Gold Antifade Mountant with DAPI, Thermo Fisher Scientific, Carlsbad, USA) and the images of miRNA signals in cells were captured by an Olympus FV1000 fluorescence microscope.

### 2.14 Animal study

The CRC cell line xenograft and the patient‐derived xenograft (PDX) models were established on 3‐ to 4‐week‐old female BALB/c nude mice (Vital River Laboratory Animal Technology Co., Ltd, Beijing, China). For CRC cell line experiments, HCT116 cells were transfected with NC and WEE1 overexpression vectors, and 2 × 10^6^ cells were deposited in each tumor. After the tumor volume reached above 100 mm^3^, which was measured by the formula V = 0.5 × L × W^2^, the mice each group (5 mice per group) were injected with 1 μM mimic or 1 μM inhibitor in the tumors and received 1 μM oxaliplatin in 200 μl of dH_2_O once every 2 days.

PDX models were established as previously reported.^12^ Briefly, fresh colorectal tumor tissues from patients were transferred to the animal center in the ice‐cold medium after the surgery. Every sample was separated into 3 × 3 × 3 mm^3^ fragments and was subcutaneously inoculated into both flanks of nude mice that were nominated as passage 1 (P1). Tumor growth was measured twice weekly. When the tumor volume was over 300 mm^3^, the mouse was anesthetized to remove the tumor, which was used to establish the next generation (P2, P3) with the same methods described above. Then, the mice carrying P3 tumors were separated into different groups to perform the experiments. Our animal study was approved by the ethics committee of SYSUCC and the Institutional Animal Care and Use Committee of SYSUCC.

### 2.15 Statistical analysis

All the experiments were repeated at least three times and the data are presented as the mean ± SD. Student's paired or unpaired tests were conducted with GraphPad Prism software (San Diego, CA, USA). Survival analyses were performed with the Kaplan‐Meier method based on the log‐rank test using statistical software (SPSS 19.0; SPSS Inc., Chicago, IL). The independent prognostic factors were identified with a Cox proportional hazard regression model. A *p* value of ˂0.05 represents statistically significance.

## 3 RESULTS

### 3.1 The expression of miR‐125b‐2‐3p in CRC tissues and the predictive value of miR‐125b‐2‐3p in CRC patients

In previous studies, we found that the expression of miR‐125b‐2‐3p was related to the prognosis and progression of CRC.^9^ Therefore, it is necessary to research the function of miR‐125b‐2‐3p in CRC to improve therapeutic efficacy and achieve long‐term survival.

**FIGURE 1:**
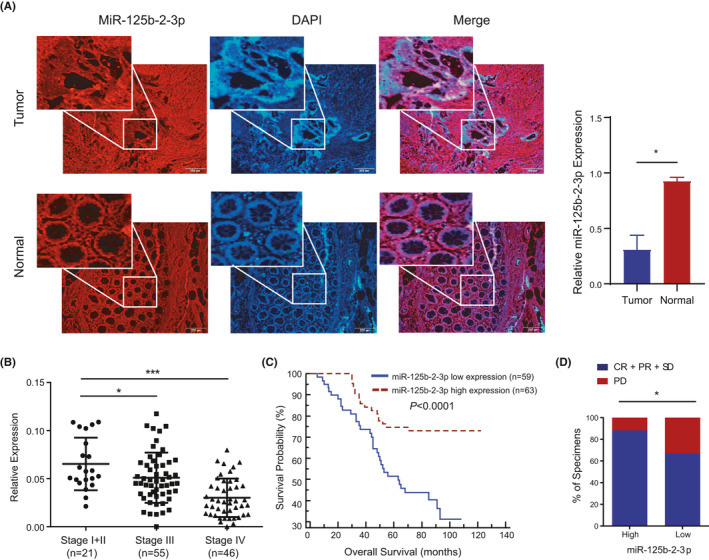
The expression of miR‐125b‐2‐3p in colorectal cancer. (A) Representative images (left) and quantification (right) of miR‐125b‐2‐3p expression in CRC tissues, specifically detected by MISH. Notably, miR‐125b‐2‐3p was expressed at low levels in cancer tissues compared to nontumor tissues. (B) Relative miR‐125b‐2‐3p levels in different clinical stages (*p* < 0.05). Stage I+II, n = 21; Stage III, n = 55; and Stage IV, n = 46. (C) The OS (*p* < 0.0001) curve of patients with high (n = 63) and low (n = 59) levels of miR‐125b‐2‐3p are shown (log‐rank test). Error bars represent the mean ±S. D. values. ^*^ *p* < 0.05, ^**^ *p* < 0.01 or ^***^ *p* < 0.001 versus the control. (D) The percentages of CRC tissues with high and low miR‐125b‐2‐3p expression were related to the DCRs. CR, complete remission; PR, partial remission; SD, stable disease; PD, progressive disease. *p < 0.05, chi‐square test

To confirm the expression of miR‐125b‐2‐3p in CRC, we used RNA fluorescence in situ hybridization (RNA‐FISH) to detect miR‐125b‐2‐3p in a CRC tissue chip by evaluating fluorescence. We found that tumor tissues showed lower fluorescence intensity than normal tissues (*p* < 0.05), indicating low expression of miR‐125b‐2‐3p in tumor tissues (Figure 1A). To assess the correlation between miR‐125b‐2‐3p and CRC progression and prognosis, we performed a quantitative real‐time PCR (qRT‐PCR) assay to investigate the expression of miR‐125b‐2‐3p in a larger cohort of CRC tissues (n = 122). The results showed that miR‐125b‐2‐3p expression was significantly reduced in advanced CRC tissues compared with early stage CRC tissues (*p* < 0.0001, Figure 1B). The patient cohort was then divided into low and high miR‐125b‐2‐3p expression groups based on the median expression level (median, 0.43) as the cut‐off value. We analyzed the overall survival (OS) of CRC patients with low or high miR‐125b‐2‐3p expression by the Kaplan‐Meier method and Cox regression analysis. As expected, the 5‐year OS of CRC patients with low miR‐125b‐2‐3p expression was significantly worse than those with high miR‐125b‐2‐3p expression (*p* < 0.0001, Figure 1C). Additionally, univariate and multivariate analyses were performed with miR‐125b‐2‐3p and other clinicopathological parameters to determine the significance of miR‐125b‐2‐3p in the prognosis and progression of CRC. Based on the univariate analysis, we found eight prognostic factors for OS, which were associated with miR‐125b‐2‐3p expression: age, gender, TNM stage, metastasis, invasion depth, lymph node metastasis, and recurrence (all *p* < 0.05, Table 1). Thus, we used multivariate Cox regression analysis to further determine the influential factors. MiR‐125b‐2‐3p was obviously an independent survival predictor for OS in CRC patients. Furthermore, we analyzed the miR‐125b‐2‐3p expression in chemosensitivity related tissue chips of CRC.^13^ In our result, we found the disease control rate (DCR) for low miR‐125b‐2‐3p expression was 66.7% (26 of 39), while that of high miR‐125b‐2‐3p expression was 88.2% (30 of 34) (Figure 1D, Figure S1B, Table S2). Thus, the patients with low expression of miR‐125b‐2‐3p showed poorer response to chemotherapy. Consequently, these results suggest that miR‐125b‐2‐3p might serve as a potential biomarker for predicting CRC prognosis and progression.

**TABLE 1:** Univariate and multivariate analysis of miR‐125b‐2‐3p associated with OS in CRC patients

Factors	Univariate analysis		Multivariate analysis	
	HR (95%)	*p* value	HR (95%)	*p* value
Age ≤60 versus >60	1.983 (1.113–3.532)	0.020		
Gender female versus male	1.999 (1.080–3.702)	0.027		
TNM stage (I‐II vs. III‐IV)	4.246 (1.321–13.646)	0.015		
Metastasis Yes. versus No.	2.963 (1.646–5.332)	<0.0001		
Tumor site Left versus right	1.253 (0.564–2.783)	0.58		
Invasion depth	1.979 (1.32–2.967)	0.001		
Differentiation (low vs. median vs. high)	1.271 (0.828–1.95)	0.272		
Lymph node metastasis Yes. versus No.	3.288 (1.774–6.093)	<0.0001	2.555 (1.365–4.780)	0.003
Recurrence, Yes. versus No.	3.933 (2.243–6.897)	<0.0001	0.488 (0.266–0.898)	0.001
miR‐125b‐2‐3p Low versus High	0.339 (0.189–0.609)	<0.0001	2.706 (1.511–4.846)	0.021

### 3.2 Overexpression of miR‐125b‐2‐3p inhibits growth and metastasis in CRC cells

**FIGURE 2:**
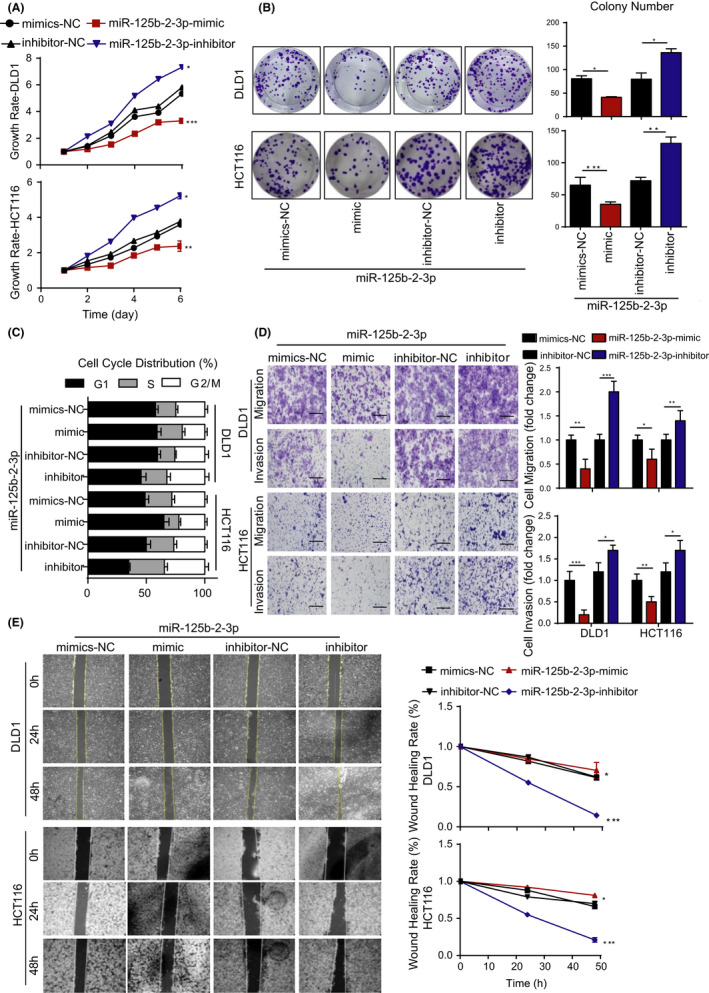
High expression of miR‐125b‐2‐3p inhibited growth and metastasis, and low expression promoted growth and metastasis. (A) Overexpression of miR‐125b‐2‐3p inhibited cell proliferation, while its downregulation promoted cell proliferation as indicated by MTS assays in CRC cells. (B) Colony formation assays show that the overexpression of miR‐125b‐2‐3p inhibited colony formation and its knockdown promoted colony formation. (C) The overexpression of miR‐125b‐2‐3p arrested the cell cycle, and its knockdown accelerated the cell cycle. (D) The migration and invasion of DLD1 and HCT116 cells were inhibited by overexpressed miR‐125b‐2‐3p but were increased by its knockdown as demonstrated by Transwell assays with and without matrix. (E) The overexpression of miR‐125b‐2‐3p inhibited cell migration, and its knockdown promoted cell migration in DLD1 and HCT116 cells as indicated by wound healing assays. Error bars represent the mean ± SD. values (n = 3). ^*^ *p* < 0.05 or ^**^ *p* < 0.01 versus the control

According to the sequence of miR‐125b‐2‐3p (UCACAAGUCAGGCUCUUGGGAC), we constructed a mimic to upregulate and an inhibitor to downregulate the expression of miR‐125b‐2‐3p (Supplementary Table S1). Treatment with the mimic upregulated the expression of miR‐125b‐2‐3p by 5‐fold or more, whereas treatment with the inhibitor downregulated the expression of miR‐125b‐2‐3p by 2.5‐fold or more in HCT116 and DLD1 CRC cells (Supplementary Figure S1A). Then, we found that the mimic decreased cell proliferation and clone formation, while the inhibitor increased cell proliferation and clone formation (Figure 2A‐B). Moreover, we analyzed the cell cycle by flow cytometry. The data showed that the G1 stage of the cell cycle was increased in the miR‐125b‐2‐3p mimic group, whereas it was decreased in the miR‐125b‐2‐3p inhibitor group (Figure 2C). Migration and invasion assays revealed that miR‐125b‐2‐3p overexpression significantly decreased the potential of metastasis in HCT116 and DLD1 cells, and downregulating miR‐125b‐2‐3p largely increased metastasis (Figure 2D). Additionally, wound healing assay showed a similar tendency as the migration and invasion assays (Figure 2E). These results validated that miR‐125b‐2‐3p overexpression attenuated tumor cell proliferation and metastasis. In contrast, the inhibition of miR‐125b‐2‐3p increased tumor proliferation and metastasis in CRC.

### 3.3 The function of miR‐125b‐2‐3p is mediated by the competing RNA lncRNA XIST

To explore how miR‐125b‐2‐3p affects the prognosis and progression of CRC, we used an online database to predict the possible influential lncRNAs. LncRNA XIST and lncRNA KCNQ1OT1, which included several binding sites on their sequences, were found. Based on our previous studies, lncRNA XIST was significantly upregulated and promoted proliferation and metastasis in CRC and gastric cancer (GC).^14^, ^15^ Thus, we decided to continue our research focusing on lncRNA XIST.

**FIGURE 3:**
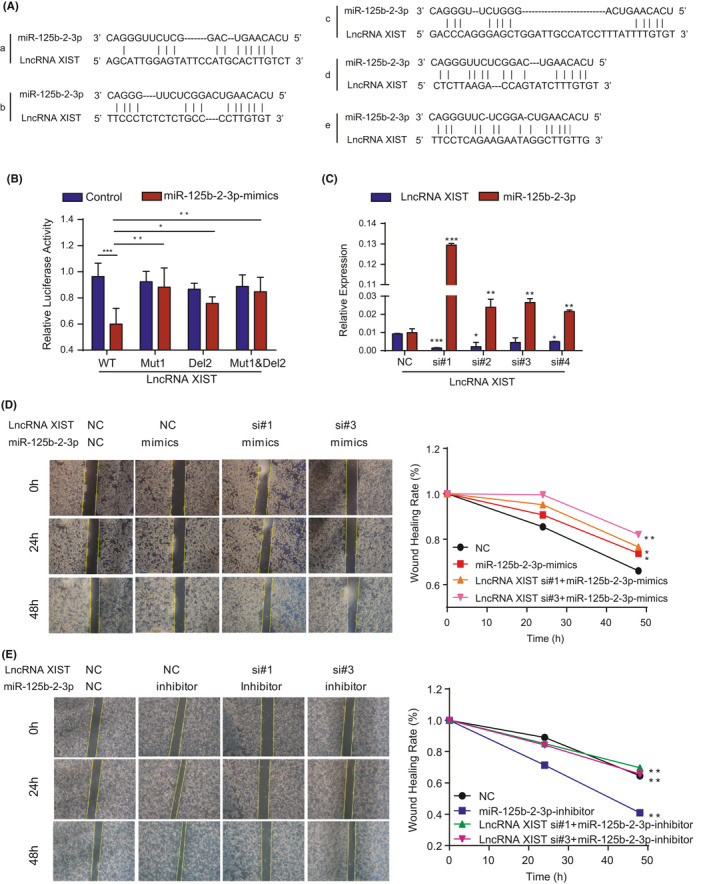
LncRNA XIST as a competing RNA mediates the function of miR‐125b‐2‐3p. (A) Schematic representation of the five predicted target sites for miR‐125b‐2‐3p in shortened lncRNA XIST. (B) Luciferase reporter assay shows that miR‐125b‐2‐3p significantly decreased the luciferase activity in XIST‐WT but not in XIST‐Mut1, XIST Mut2, and XIST Mut1 & Mut2 in HCT116 cells. (C) The relative expression of lncRNA XIST and miR‐125b‐2‐3p in lncRNA XIST‐knockdown cells. (D) The overexpression of miR‐125b‐2‐3p inhibited cell migration and knockdown of lncRNA XIST increased the inhibition rate as indicated by the wound healing assay. (E) The downregulation of miR‐125b‐2‐3p promoted cell migration, and the knockdown of lncRNA XIST restored migration, as indicated by the wound healing assay. Error bars represent the mean ± SD. values (n = 3). ^*^ *p* < 0.05 or ^**^ *p* < 0.01 versus the control

First, we analyzed the miR‐125b‐5p pool to predict the interacting long non‐coding RNAs. Since miR‐125b‐2‐3p and miR‐125b‐5p contained similar mature sequences, miR‐125b‐2‐3p lacked mature database. With TargetScan algorithms, we identified four potential miR‐125b‐5p binding sites in the CDS region of lncRNA XIST. However, the sequence of lncRNA XIST was too long to construct into one vector, thus we chose a shortened sequence of lncRNA XIST (3097 bp) that contained two closet sites (Supplementary Figure S2A). In addition, we found five predicted sites of miR‐125b‐2‐3p in the shortened sequence of lncRNA XIST (Figure 3A). Then, we analyzed the secondary structure of lncRNA XIST to detect the exact binding locations of miR‐125b‐5p and miR‐125b‐2‐3p (Supplementary Figure S2B). To determine whether lncRNA XIST could directly bind to miR‐125b‐5p and miR‐125b‐2‐3p by competitively combining with a miRNA response element (MRE), we used the two predicted sites of miR‐125b‐5p to construct dual‐luciferase reporters gene plasmids containing wild type (WT), mutation 1 (Mut1), mutation 2 (Mut2) or mutation 1&2 (Mut1&Mut2) lncRNA XIST (Figure S2C). The ratio between firefly and renilla luciferase has been calculated on the simple diagram (Figure S2D), and our results showed that the miR‐125b‐5p mimic obviously inhibited lncRNA XIST expression in WT lncRNA XIST. In contrast, miR‐125b‐5p mimic failed to influence both mutation sites. Also, miR‐125b‐2‐3p inhibited lncRNA XIST expression in all groups (Supplementary Figure S3E). Therefore, these results suggest that miR‐125b‐5p could directly bind to predicted sites 1 and 2 on lncRNA XIST, and miR‐125b‐2‐3p could obviously bind to lncRNA XIST. For further confirmation, we chose the two closest predicted sites of miR‐125b‐2‐3p in the former sequence to construct luciferase reporters containing wild type (WT), mutation 1 (Mut1), deletion 2 (Del2) or mutation 1& deletion2 (Mut1&Del2) lncRNA XIST. The data showed that the miR‐125b‐2‐3p mimic obviously inhibited lncRNA XIST expression in WT lncRNA XIST. Conversely, miR‐125b‐2‐3p mimic failed to influence the mutation site and deletion site (Figure 3B). Furthermore, we designed four short‐interfering RNAs (siRNAs) aimed at lncRNA XIST to transfect CRC cells. After knocking down lncRNA XIST, miR‐125b‐2‐3p was clearly upregulated (Figure 3C), indicating that miR‐125b‐2‐3p levels were controlled by lncRNA XIST with direct binding. To validate the potential influence of lncRNA XIST on the function of miR‐125b‐2‐3p, we cotransfected miR‐125b‐2‐3p mimic and lncRNA XIST si#1 or si#3 and performed a wound‐healing assay. The data verified that lncRNA XIST significantly decreased the wounding healing rate when miR‐125b‐2‐3p was overexpressed (Figure 3D). Conversely, downregulation of miR‐125b‐2‐3p increased the wound healing rate and could be counteracted by knocking down lncRNA XIST (Figure 3E). Therefore, these results indicated that lncRNA XIST could regulate miR‐125b‐2‐3p function as a ceRNA.

### 3.4 MiR‐125b‐2‐3p exerts its function by influencing the target gene WEE1

**FIGURE 4:**
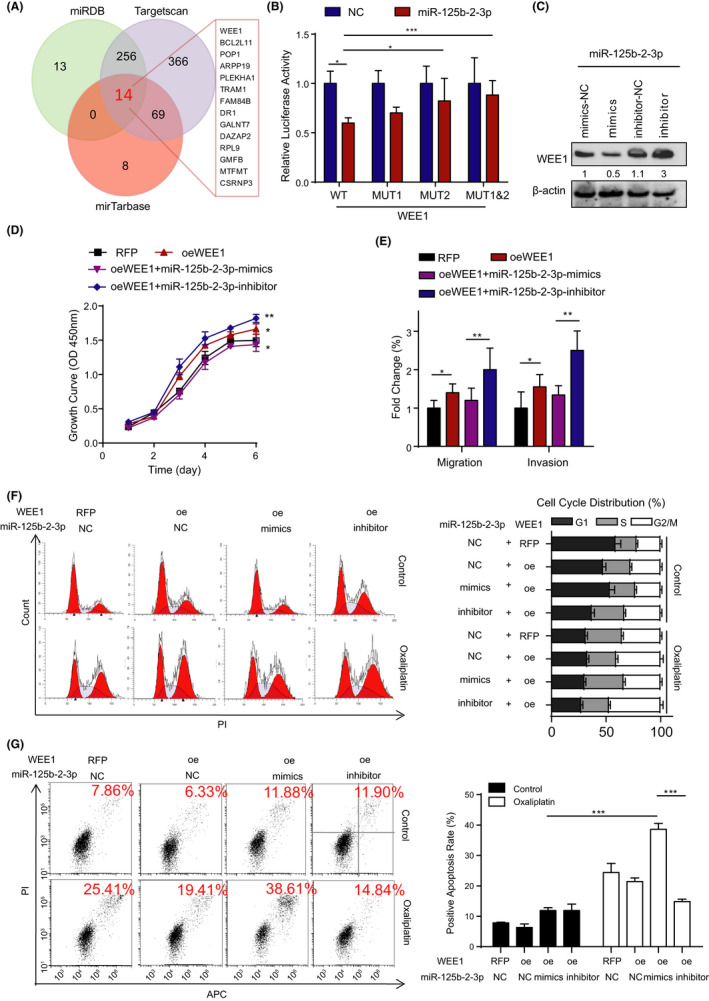
MiR‐125b‐2‐3p exerts its function by influencing the target gene WEE1. (A) Venn diagram showing the overlap among the target genes from miRDB, TargetScan, and miRTarBase. (B) Luciferase reporter assay shows that miR‐125b‐2‐3p significantly decreased the luciferase activity in WEE1‐WT but not in WEE1‐Mut2 and WEE1‐Mut1&Mut2 in HCT116 cells. (C) Western blot of WEE1 in the total lysates of HCT116 cells transfected with the miR‐125b‐2‐3p mimic, inhibitor or control siRNA. (D) The cell proliferation was measured in HCT116 cells transduced with WEE1 lentivirus and the mimic or inhibitor of miR‐125b‐2‐3p. (E) Representative images (left) and quantification (right) of the migration and invasion assays of the CRC cells transduced with WEE1 lentivirus and the mimic or inhibitor of miR‐125b‐2‐3p. (F) Representative images (left) and quantification (right) of the cell cycle in the CRC cells transduced with WEE1 lentivirus and the mimic or inhibitor of miR‐125b‐2‐3p treated with or without 10 µM oxaliplatin for 48 h; the cell cycle was determined using the flow cytometry. (G) Representative images (left) and quantification (right) of cell viability in the indicated cells transduced with WEE1 lentivirus and the mimic or inhibitor of miR‐125b‐2‐3p treated with or without 30 µM oxaliplatin for 48 h; cell apoptosis was determined using the flow cytometry. Data are presented as the mean ± SD. (n = 3). ^*^ *p* < 0.05 or ^**^ *p* < 0.01 versus the control

MiR‐125b‐2‐3p is an ncRNA that exerts its regulatory function through targeting downstream genes. To explore target genes, we analyzed three databases (miRDB, TargetScan, and miRTarBase), and the resulting Venn diagram showed 14 target genes. Interestingly, WEE1, which belongs to the cell cycle pathway and contributes to the G_2_‐M checkpoint, was indicated in the Venn diagram (Figure 4A). In addition, according to the Gene Expression Profiling Interactive Analysis (GEPIA) database, WEE1 was obviously upregulated in CRC compared with that in normal tissues (Supplementary Figure S3A), which was coincident with our research. Thus, we hypothesized that WEE1 may be the target gene of miR‐125b‐2‐3p.

As is well known, miRNAs can bind to the 3’‐untranslated regions (3’‐UTRs) of protein‐coding genes regulating their expression. According to the analysis of TargetScan algorithms, two potential miR‐125b‐2‐3p binding sites were identified in the WEE1 3’‐UTR (Supplementary Figure S3B). Then, we introduced one mutation in each potential binding site of the WEE1 3’‐UTR, which disrupted the base repair with the miR‐125b‐2‐3p seed sequence (Supplementary Figure S3B). As performed in the former methods, we tested the dual‐luciferase reporter activity in CRC cells. The outcomes showed that the miR‐125b‐2‐3p mimic significantly inhibited WEE1 transcriptional expression in the WEE1 3’‐UTR WT and the Mut 1 group. However, the miR‐125b‐2‐3p mimic did not affect its expression in the WEE1 3’‐UTR Mut 2 group (Figure 4B). These results strongly indicated that miR‐125b‐2‐3p could directly bind to WEE1. The miR‐125b‐2‐3p mimic inhibited WEE1 expression at the protein level, and the inhibitor could instead promote WEE1 expression (Figure 4C).

To confirm the impact of miR‐125b‐2‐3p on the function of WEE1, we further investigated how WEE1 influenced the development of CRC cells. First, WEE1 overexpression promoted growth in CRC cells, and the miR‐125b‐2‐3p mimic restored partial growth ability after WEE1 was overexpressed. The proliferation rates could be accelerated largely through the transfection of WEE1 overexpression vectors and the miR‐125b‐2‐3p inhibitor (Figure 4D). Additionally, the results of migration and invasion showed a similar tendency (Figure 4E). WEE1 kinase seemed to be a G_2_‐M checkpoint regulator; thus, the overexpression of WEE1 increased the distribution of the G_2_‐M stage of the cell cycle. After treatment with the miR‐125b‐2‐3p mimic in the WEE1 overexpression cell lines, the G_2_‐M distribution decreased significantly, while its inhibitor further increased the G_2_‐M distribution. Based on our previous study,^9^ the expression of miR‐125b‐2‐3p was related to the sensitivity of CRC to first‐line chemotherapy. Thus, we treated cells with oxaliplatin and found that tumor cells were arrested at the G_2_‐M stage, which allowed for DNA repair (Figure 4F). The inhibition of WEE1 reduced G_2_‐M arrest, which has been reported to lead to premature entry into mitosis followed by cell death through mitotic catastrophe or apoptosis.^16^, ^17^, ^18^, ^19^, ^20^ Therefore, we performed an apoptosis assay in the former group. Interestingly, we discovered that the WEE1 overexpression group protected cells from DNA damage by oxaliplatin, and the miR‐125b‐2‐3p mimic plus WEE1 overexpression group showed an increased amount of apoptotic cells. In contrast, in the inhibitor plus WEE1 group, the apoptosis rate of CRC cells was largely decreased (Figure 4G). These data suggest that high miR‐125b‐2‐3p expression increased drug sensitivity to CRC cells. Conversely, the low expression of miR‐125b‐2‐3p increased the resistance of cells to chemotherapeutic medicine.

### 3.5 Overexpression of miR‐125b‐2‐3p increases chemotherapeutic sensitivity by targeting WEE1 in vivo

To deeply investigate the relationship between miR‐125b‐2‐3p and WEE1 in CRC proliferation in vivo, HCT116 cells were transfected with WEE1 vectors (oeWEE1) or red fluorescent protein (RFP) as a control and were subcutaneously inoculated into nude mice. After 1 week (the tumors were measurable), the nude mice inoculated with WEE1 overexpression HCT116 cells were averagely separated into three groups: one group was treated with control, another was injected with miR‐125b‐2‐3p agomir, and the other was injected with miR‐125b‐2‐3p antagomir. Furthermore, each group was randomly assigned into two groups that received treatment with or without oxaliplatin for 3 weeks. In the control group, the WEE1 overexpression plus miR‐125b‐2‐3p agomir subgroup dramatically suppressed the growth of the tumor xenografts, and WEE1 overexpression plus miR‐125b‐2‐3p antagomir subgroup showed an obvious promotion of tumor growth, which was faster than that of the WEE1 overexpression subgroup (Supplementary Figure S4A). After treatment with oxaliplatin, the proliferation of only the subgroup of oeWEE1 plus agomir was significantly inhibited (*p* < 0.001) (Supplementary Figure S4B‐D). These results showed that the high expression of WEE1 promoted growth and that the miR‐125b‐2‐3p mimic repressed the growth‐promoting effect of WEE1. Furthermore, the miR‐125b‐2‐3p mimic enhanced the sensitivity of CRC to oxaliplatin.

**FIGURE 5:**
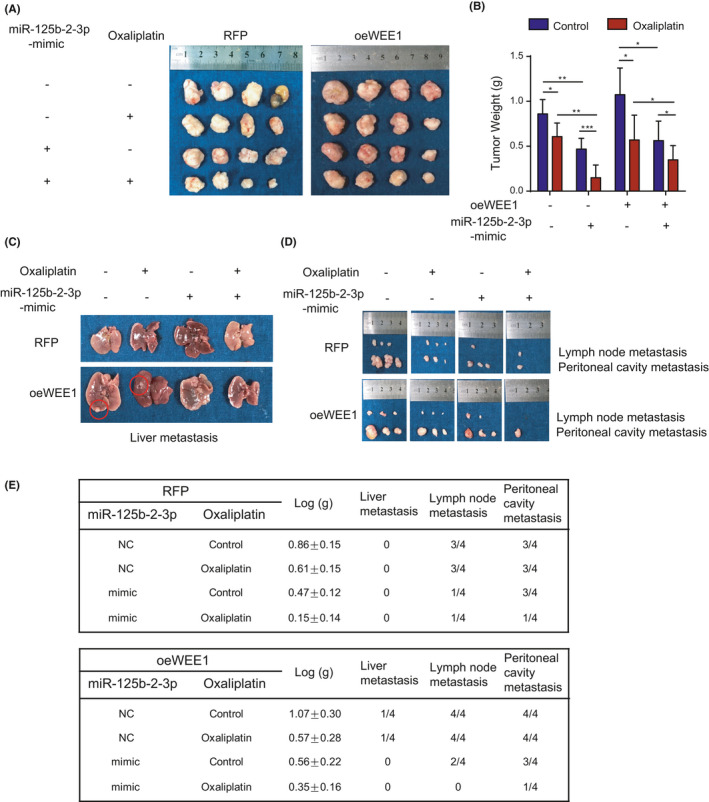
In vivo, miR‐125b‐2‐3p targets WEE1 to influence the function of colorectal cancer cells. (A) The tumors of each group in situ were extracted and recorded. (B) The tumors of each group were weighted and compared. (C) The livers were extracted and the metastases were recorded. (D) Lymph node metastasis and peritoneal cavity metastasis were extracted and recorded. (E) The records of animal experiments on the primary tumors, liver metastasis, lymph metastasis, and peritoneal cavity metastasis in situ. Data are presented as the mean ± SD. (n = 4). ^*^ *p* < 0.05 or ^**^ *p* < 0.01 versus the control

To analyze the influence of miR‐125b‐2‐3p on tumor metastasis, we constructed a model in situ. RFP and WEE1 overexpression tumors separated from subcutaneously inoculated tumors were averagely divided into small pieces of similar size and tied to the base of the cecum. After 1 week, each group was equally divided into two subgroups, and then one of the RFP groups and one of the oeWEE1 groups were treated with agomir through intraperitoneal injection, resulting in the following groups: (1) the RFP control group; (2) the RFP group with miR‐125b‐2‐3p agomir; (3) the oeWEE1 control group; and (4) the oeWEE1 group with miR‐125b‐2‐3p agomir. Each group of mice received with or without oxaliplatin for 4 weeks. In the end, all mice were euthanized, whose tumors were all separated from the in situ and weighed (Figure 5A‐B). Simultaneously, the numbers of metastases in the liver, lymph node, and peritoneal cavity were counted (Figure 5C‐D). The results showed that the groups with the miR‐125b‐2‐3p agomir had obviously decreased tumor sizes and numbers of metastatic tumors. The oeWEE1 group had increased tumor sizes in situ and numbers of distanced tumor metastases. Treatment with oxaliplatin could not prevent the metastatic processes in the WEE1 overexpression group. However, after an injection of the miR‐125b‐2‐3p mimic, we found that there was no liver metastasis Besides, the metastatic lymph nodes were smaller, and the peritoneal cavity metastases were much less. Moreover, we have not found any liver or lymph node metastasis, and there were only small peritoneal cavity metastases in the oeWEE1 plus miR‐125b‐2‐3p mimic group treated with oxaliplatin (Figure 5E).

### 3.6 Competing lncRNA XIST mediates the function of WEE1 by influencing miR‐125b‐2‐3p

**FIGURE 6:**
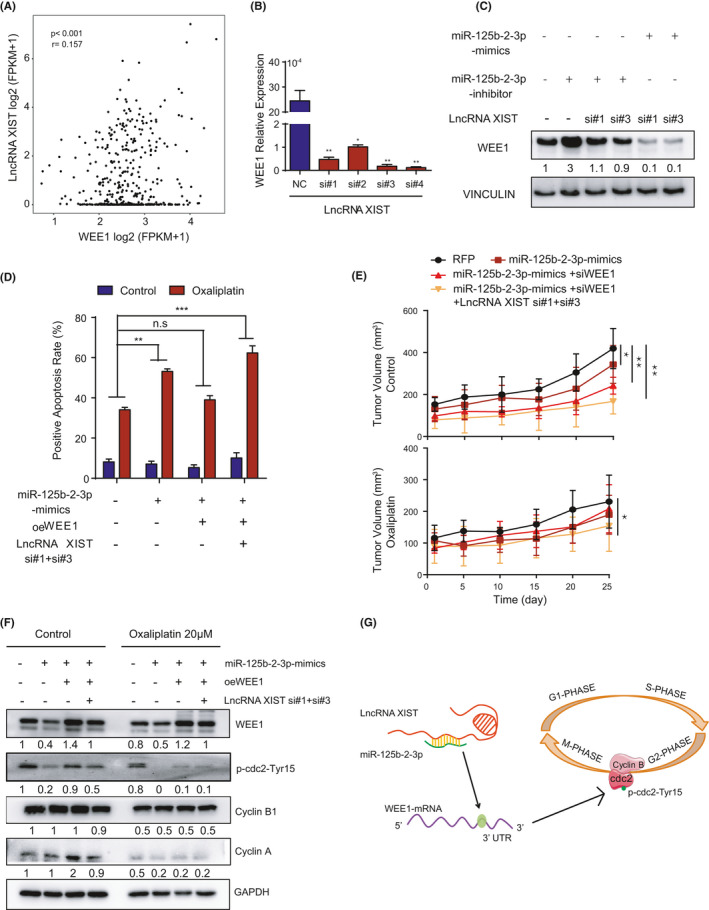
Competing lncRNA XIST mediates the function of miR‐125b‐2‐3p by targeting WEE1. (A) Correlation of WEE1 and lncRNA XIST. The *r* values and *P* values are from Pearson's correlation analysis. (B) Real‐time qPCR quantification of WEE1 after the knockdown of lncRNA XIST. (C) Western blot of WEE1 in the total lysates of HCT116 cells transfected with miR‐125b‐2‐3p mimic, inhibitor, lncRNA XIST si#1, lncRNA XIST si#3 or control siRNA. (D) Representative images (left) and quantification (right) of cell viability in the indicated cells treated with or without 30 µM oxaliplatin for 48 h. The cells were transduced with the WEE1 lentivirus, mimic of miR‐125b‐2‐3p, or with si#1+si#3 to knock down lncRNA XIST; the cell apoptosis was measured with flow cytometry (n = 3). (E) PDX tumors were treated with or without oxaliplatin, and the tumor volumes were measured at each time point (n = 5). (F) WEE1 protein levels in HCT116 cells following the ectopic expression of miR‐125b‐2‐3p and/or knockdown of lncRNA XIST and/or overexpressed WEE1. (G) Proposed working model of this study. MiR‐125b‐2‐3p was absorbed by lncRNA XIST and regulated the expression of the WEE1, thus influencing the cell cycle. Data are presented as the mean ± SD. ^*^ *p* < 0.05 or ^**^ *p* < 0.01 versus the control

To determine the relationship between WEE1 and lncRNA XIST, we first analyzed databases using the University of California at Santa Cruz (UCSC) Xena Browser. The data confirmed that WEE1 and lncRNA XIST had an apparent positive correlation (Figure 6A). Second, the expression of WEE1 decreased considerably when lncRNA XIST was downregulated (Figure 6B). Besides, we found that WEE1 expression was increased following the inhibition of miR‐125b‐2‐3p. The knockdown of lncRNA XIST might release miR‐125b‐2‐3p, resulting in miR‐125b‐2‐3p being correspondingly upregulated; thus, WEE1 expression was significantly decreased. The lowest expression of WEE1 was observed in the group with knocked down lncRNA XIST and overexpressed miR‐125b‐2‐3p (Figure 6C). Then, an apoptosis assay demonstrated that cells with miR‐125b‐2‐3p overexpression were relatively more sensitive to oxaliplatin, and ectogenic WEE1 in miR‐125b‐2‐3p overexpressed cells induced partial resistance. Finally, knockdown of lncRNA XIST produced the most obvious sensitivity to oxaliplatin (Figure 6D). In addition, PDX models showed a similar tendency in vivo. Four siRNAs were designed to degrade the protein level of WEE1 (Supplementary Figure S5D). To knock down the expression of WEE1 by direct injection into the tumor in PDX models, the fourth siRNA with higher efficiency of knockdown was further modified with methylation and cholesterol to specifically target tumor cells in vivo. Then we performed multipoint injection of the RNAi solution into the tumor directly (1 nmol for each tumor, twice per week). The overexpression of miR‐125b‐2‐3p slowed tumor growth and increased drug sensitivity; moreover, knocking down WEE1 further slowed growth and promoted drug sensitivity, finally when miR‐125b‐2‐3p was overexpressed, lncRNA XIST was knocked down and WEE1 knocked down simultaneously. it conferred the slowest tumor growth and the highest drug sensitivity among all the groups (Figure 6E, Supplementary Figure S5B‐C). In addition, the weight of the mice did not change after treatment with oxaliplatin (Supplementary Figure S5A). Overexpression of miR‐125b‐2‐3p and/or knockdown of lncRNA XIST considerately reduced WEE1 protein, while overexpression of WEE1 restored its protein level in CRC cells. By interfering WEE1 level, the cell cycle could be influenced largely through the activation of p‐cdc2Tyr15, and the treatment of oxaliplatin decreased the expression of cyclin B and cyclin A (Figure 6F‐G).

### 3.7 **Overexpression of miR‐125b‐2‐3p can restore the sensitivity of drug‐resistant CRC cells**.

**FIGURE 7:**
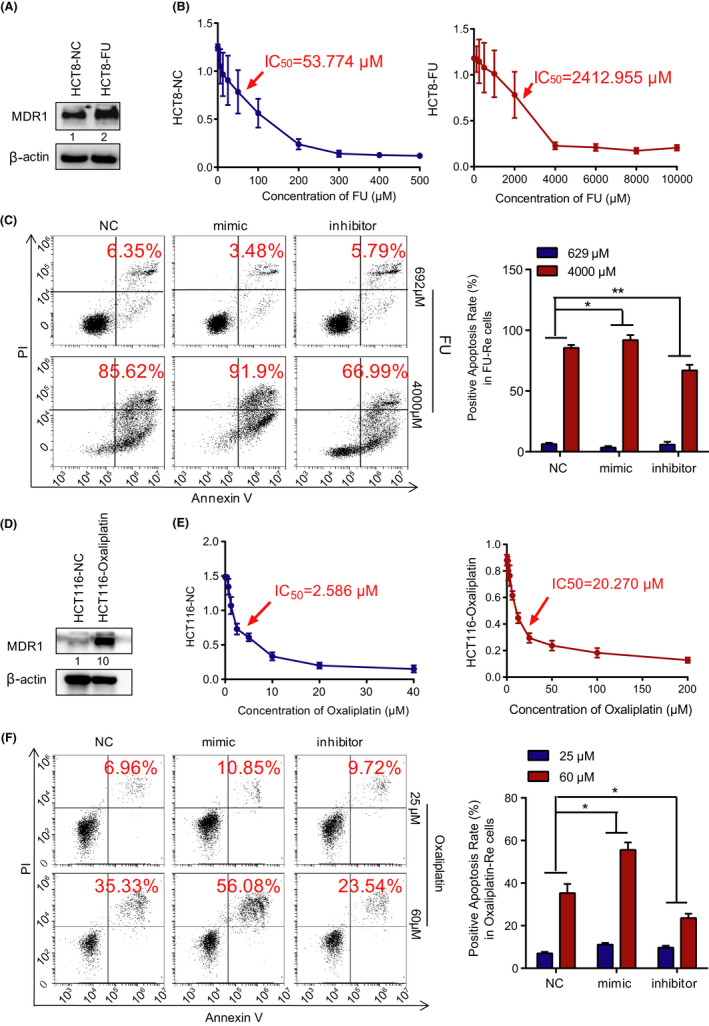
MiR‐125b‐2‐3p overexpression restores the sensitivity of drug‐resistant colorectal cancer cells. (A) Western blot of MDR1 in the total lysates of HCT8 normal cells and drug‐resistant cells. (B) MTS assays show the viability of HCT8 normal cells and HCT8 drug‐resistant cells exposed to 5‐fluorouracil (72 h). (C) Images (left panel) and quantification (right panel) of apoptosis assays of the indicated cells cultured with 692 μM or 4,000 μM 5‐fluorouracil for 2 days. (D) Western blot of MDR1 in the total lysates of HCT116 normal cells and drug‐resistant cells. (E) MTS assays show the viability of HCT116 normal cells and HCT116 drug‐resistant cells exposed to oxaliplatin (72 h). (F) Images (left panel) and quantification (right panel) of the apoptosis assays of the indicated cells cultured with 25 μM or 60 μM oxaliplatin for 2 days. Data are presented as the mean ± SD. (n = 3). ^*^ *p* < 0.05 or ^**^ *p* < 0.01 versus the control

To expand the clinical applications, we also cultured the fluorouracil‐resistant (FU‐Re) CRC cell line HCT8 (HCT8‐FU) and oxaliplatin‐resistant (Oxaliplatin‐Re) CRC cell line HCT116 (HCT116‐Oxaliplatin) to detect the effect of drug resistance on clinical treatments. ATP‐binding cassette superfamily transporter genes, such as MDR protein (MRP) and P‐glycoprotein/multidrug resistance 1 (MDR1) are usually upregulated in CRC. And they often influence the responses of malignant cells to certain anticancer chemotherapeutic agents. Our data confirmed that MDR1 expression was higher in HCT8‐FU cells than in HCT8 control cells (Figure 7A). The IC_50_ value of HCT8‐FU cells was 2,412.955 μM, which was 45‐fold higher than that of HCT8 normal cells (IC_50_ = 53.774 μM) (Figure 7B). Furthermore, we used HCT8‐FU cells to analyze the positive apoptosis rate in overexpressed and inhibited miR‐125b‐2‐3p with different FU concentrations. The mimic group could obviously restore chemosensitivity in drug‐resistant cells. Conversely, the inhibitor group enhanced resistance to chemotherapy in drug‐resistant CRC cells (Figure 7C). Additionally, we used HCT116‐Oxaliplatin cells to confirm the former findings. MDR1 was also highly expressed in oxaliplatin‐Re HCT116 cells compared with that in HCT116 normal cells (Figure 7D). Oxaliplatin‐Re HCT116 had higher resistance (IC_50_ = 20.270 μM) than control cells (IC_50_ = 2.586 μM) by approximately 10‐fold (Figure 7E). Furthermore, the miR‐125b‐2‐3p mimic group significantly restored the drug sensitivity of oxaliplatin, and its inhibitor group dramatically increased drug resistance (Figure 7F). Consequently, miR‐125b‐2‐3p could reverse the chemotherapeutic resistance of CRC cells, and this finding might be significant for clinical treatments for CRC.

## 4 DISCUSSION

Currently, low chemotherapeutic efficiency and drug resistance remain the most substantial problems in clinical treatments, especially for advanced CRC patients, who might mainly depend on chemotherapeutic treatments. Thus, promoting drug efficiency and reducing drug resistance would help many CRC patients. In previous studies, we confirmed that miR‐125b‐2‐3p was lowly expressed in advanced CRC compared with normal tissues, which could serve as an independent prognostic factor for predicting disease progression and evaluating the efficiency of chemotherapy.^9^ Based on these results, we postulated that miR‐125b‐2‐3p may contain an expression pattern related to drug sensitivity and can act as a potential biomarker for chemotherapy in CRC.

There are many relevant reports that microRNAs are involved in predicting the progression and prognosis of CRC. For example, Zhang Y *et al*. reported that microRNA‐494 could be a potential prognostic marker and therapeutic target, and could limit CRC cell proliferation and tumorigenesis by inducing the Wnt/ß‐catenin pathway.^21^ Junjie Xiao et al. found that inhibiting miR‐4260 might be a potential therapeutic strategy by influencing MCC and SMAD4 genes in CRC.^22^ In addition, microRNA‐181a was able to promote angiogenesis by targeting SRCIN1 in CRC.^23^ In recent researches, microRNAs in peripheral blood, such as circulating microRNA‐1290,^24^ plasma miR‐200c,^25^ miR‐21,^26^ and miR‐203,^27^ were crucial for the early detection and predicting prognosis in CRC.^28^, ^29^ Additionally, a few results have shown that some microRNAs alter drug sensitivity in various tumors. Evidently, the miR‐125b family consists of members with controversial properties in different cancer types; they may play important roles in the development of cancers.^30^, ^31^, ^32^, ^33^ Here, our study primarily reported that miR‐125b‐2‐3p was related to chemotherapeutic sensitivity. This is the first study to explore the involvement of the miR‐125 family in the chemosensitivity of CRC.

LncRNA XIST is an essential lncRNA required for X‐chromosome inactivation, interacting with 81 proteins involved in the nuclear matrix, chromatin modification, and RNA remodeling pathways.^34^ In recent years, lncRNA XIST has been reported to be strongly associated with the development and progression of cancers. Our previous studies on the function of lncRNA XIST in gastrointestinal cancers showed that lncRNA XIST was a meaningful biomarker to predict prognosis in CRC and gastric cancer, which was also confirmed by DL. Chen et al.^14^, ^15^ In our data, we primarily reported that lncRNA XIST could influence miR‐125b‐2‐3p, resulting in a change in drug sensitivity. Since lncRNA XIST was upregulated in CRC compared with normal tissues, we speculated that higher expression of lncRNA XIST might lead to lower miR‐125b‐2‐3p expression, thus impacting the effect of chemotherapy. Finally, we obtained many targeted sites with the help of bioinformatic analysis. However, we used only one sequence of lncRNA XIST that contained two of the closest binding sites to miR‐125b‐2‐3p; therefore, there might be other potential sites that could also be targeted.

WEE1 is a nuclear kinase regulating cell cycle progression.^35^, ^36^ Many reports have confirmed that WEE1 is highly expressed in the diversity of tumor types ^37^ and is associated with poor outcomes,^16^, ^38^, ^39^ but its expression has not been clarified in CRC.^16^, ^40^ In recent years, WEE1 has emerged as a promising target in cancer treatments.^36^, ^41^ Furthermore, MK‐1775/AZD1775, a WEE1 inhibitor, is being tested in clinical trials.^35^ MK‐1775/AZD1775 could obviously interfere S phase and G2/M phase in the cell cycle, leading to the inhibition of WEE1 and ATR kinases producing synthetic lethality and suppressing metastasis.^42^ Our data showed a similar tendency where overexpression of miR‐125b‐2‐3p decreased the expression of WEE1, thus CDC2 was activated by dephosphorylation at Tyr15. Cdc2/cyclin B complex mainly controls the G2/M phase checkpoint. In the G2/M phase, cyclinA was gradually degraded and was replaced by cyclinB. Therefore, activation of Cdc2/CyclinB could promote the cells to enter into the M phase. In that case, chemotherapeutic treatments could result in a downregulation in the distribution of the S‐phase and G2/M‐phase. Subsequently, treatments made the cells with unrepaired or under‐replicated DNA easier to enter into mitosis and induce mitotic catastrophe. Finally, these mechanisms confirmed our hypothesis that CRC cells with higher expression of miR‐125b‐2‐3p would be more sensitive to chemotherapy. Various cell cycle regulators and proteins have been studied for several years, but their clinical utilities are still debated in the treatment of CRC. We speculated that the lack of sensitive markers might be an important factor. In this study, we also found that miR‐125b‐2‐3p could directly bind to WEE1 and regulate its expression; thus, miR‐125b‐2‐3p might be a significant biomarker to predict the curative effects of targeting WEE1.

Last but not least, overexpressing miR‐125b‐2‐3p was verified to reverse drug resistance. However, we could not clarify which specific mechanisms are obviously changed in influencing chemotherapeutic sensitivity. Therefore, future studies on lncRNA XIST/miR‐125b‐2‐3p/WEE1 axis to focus on changes in drug sensitivity or resistance are imminently needed.

In conclusion, we determined that miR‐125b‐2‐3p expression was considerably decreased in CRC, especially in advanced stages, and predicted chemotherapeutic sensitivity and prognosis in CRC patients. Functional experiments showed that downregulated miR‐125b‐2‐3p expression promoted cell development and metastasis in vivo and in vitro, and vice versa. Simultaneously, a high miR‐125b‐2‐3p level increased drug sensitivity, and low miR‐125b‐2‐3p expression decreased drug sensitivity. Furthermore, we demonstrated that lncRNA XIST could absorb miR‐125b‐2‐3p affecting its function. Moreover, miR‐125b‐2‐3p exhibited its regulatory function by binding to the targeted gene WEE1. Our results provide a new regulatory axis, lncRNA XIST/miR‐125b‐2‐3p/WEE1 axis that influences drug sensitivity in CRC. Therefore, miR‐125b‐2‐3p may be a novel prognostic and drug sensitivity marker for CRC treatments.

## 5 ETHICS APPROVAL AND CONSENT TO PARTICIPATE

The clinical CRC specimens were conducted with permission by the Institutional Research Ethics Committee of Sun Yat‐sen University Cancer Center, China. All animal experiments were performed in accordance with a protocol approved by the Ethics Committee of the Institutional Animal Care of Sun Yat‐sen University Cancer Center, China.

## 6 CONSENT FOR PUBLICATION

The content of this manuscript has not been previously published and is not under consideration for publication elsewhere.

## CONFLICT OF INTEREST

The authors declare no conflicts of interest.

## AUTHORS CONTRIBUTIONS

Conception and design: J.‐H. Lu, Z.‐L. Zeng and R.‐H. Xu; Development of methodology: Y. Wang, Y.‐N. Wang, Z.‐H.‐Chen, Q.‐N. Wu, D.‐D. Yang, H.‐Q. Ju; Acquisition of data (provided animals, acquired and managed patients, provided facilities, etc.): J.‐H. Lu, Y. Wang, H. Sheng, Y.‐N. Wang, Z.‐H.‐Chen, Q.‐N. Wu; Analysis and interpretation of data (e.g., statistical analysis, biostatistics, computational analysis): Y.‐X Chen, K. Yu, Z.‐X. Chen; Writing, review, and/or revision of the manuscript: J.‐H. Lu, J.‐B. Zheng. R.‐H. Xu and Z.‐L. Zeng; Administrative, technical, or material support (i.e., reporting or organizing data, constructing databases): P.‐S. Hu, H. Sheng, H.‐Y. Mo and J.‐J Hu; Study supervision: R.‐H. Xu and Z.‐L. Zeng; Comments and suggestions: H.‐Q. Ju.

## Supporting information

Fig S1

Fig S2

Fig S3

Fig S4

Fig S5

Table S1

Table S2

Supplementary Material

## Data Availability

The datasets used and/or analyzed during the current study are available from the corresponding author on reasonable request.
