# Supplementary figures and images for: The lncRNA XIST/miR‐125b‐2‐3p axis modulates cell proliferation and chemotherapeutic sensitivity via targeting Wee1 in colorectal cancer
Source: Cancer Med. 2021 Mar 5;10(7):2423–41. doi: 10.1002/cam4.3777 (PMC7982616; doi:10.1002/cam4.3777)

A

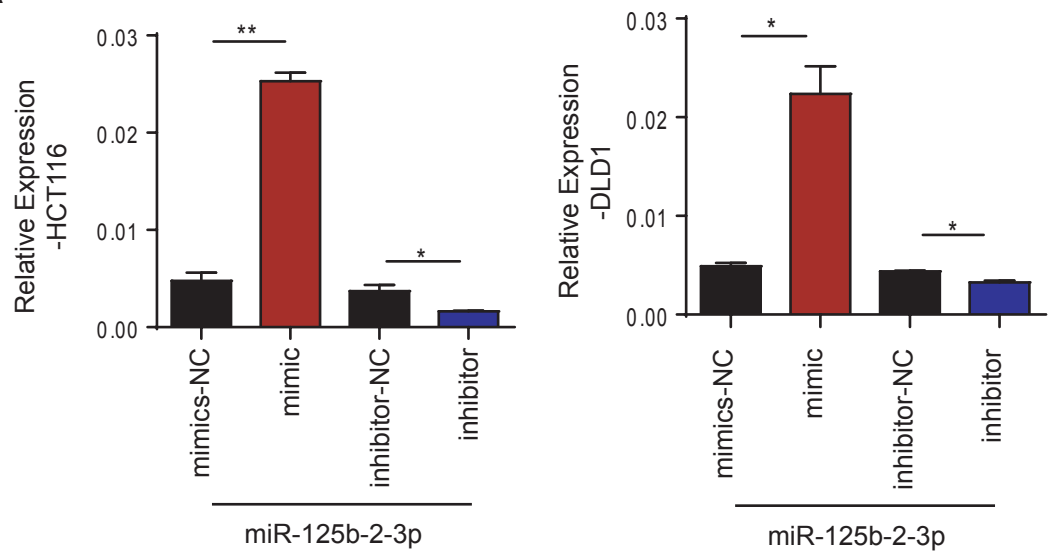

B

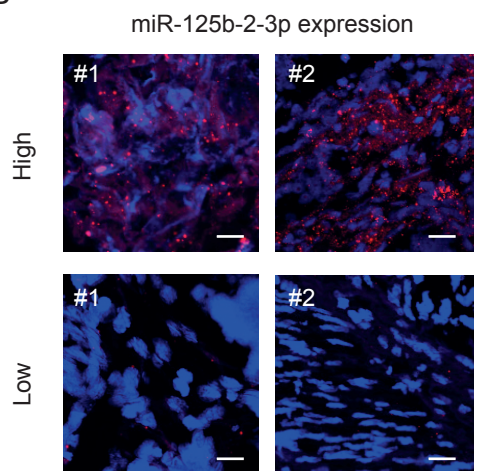

Supplement: Supplementary file 1 — Fig S1 [file CAM4-10-2423-s005.pdf]

A

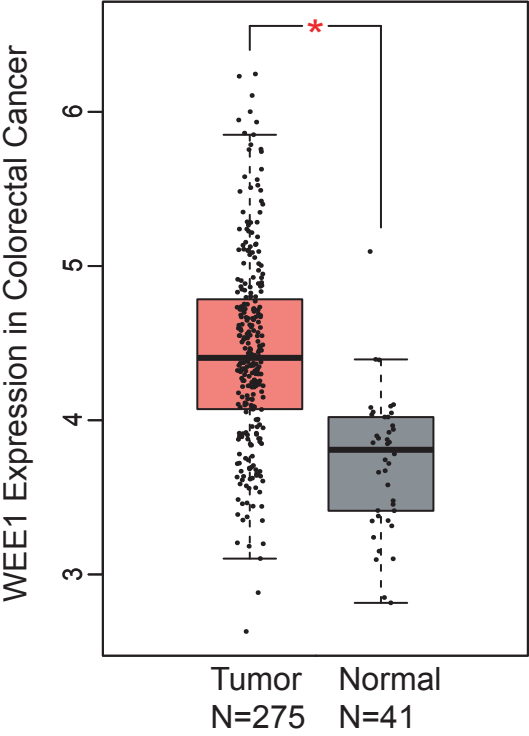

B

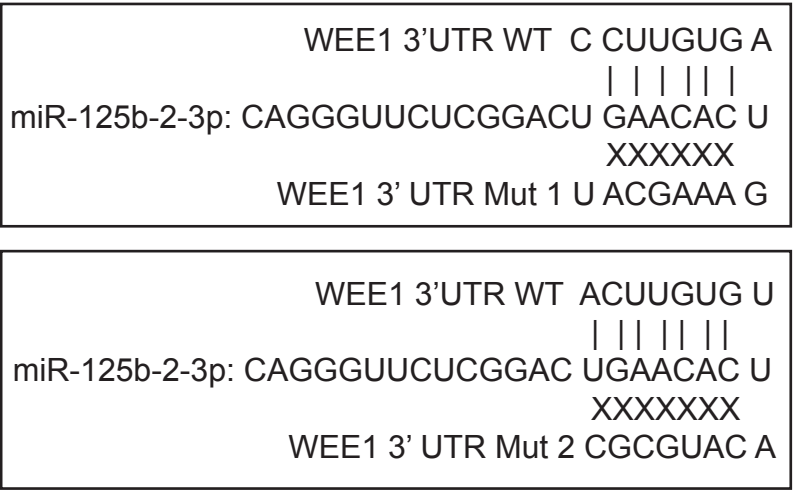

Supplement: Supplementary file 3 — Fig S3 [file CAM4-10-2423-s004.pdf]

A

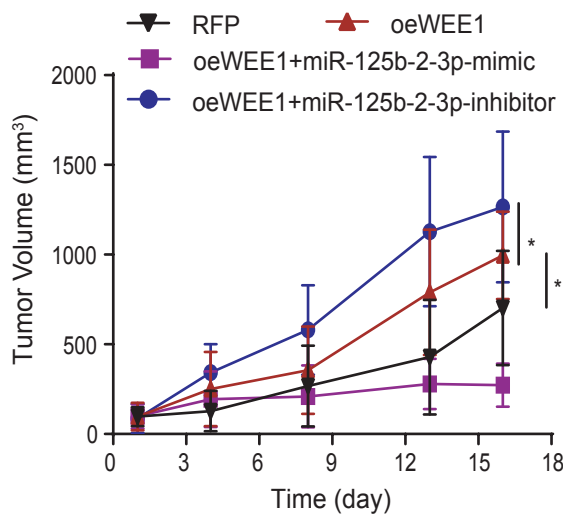

B

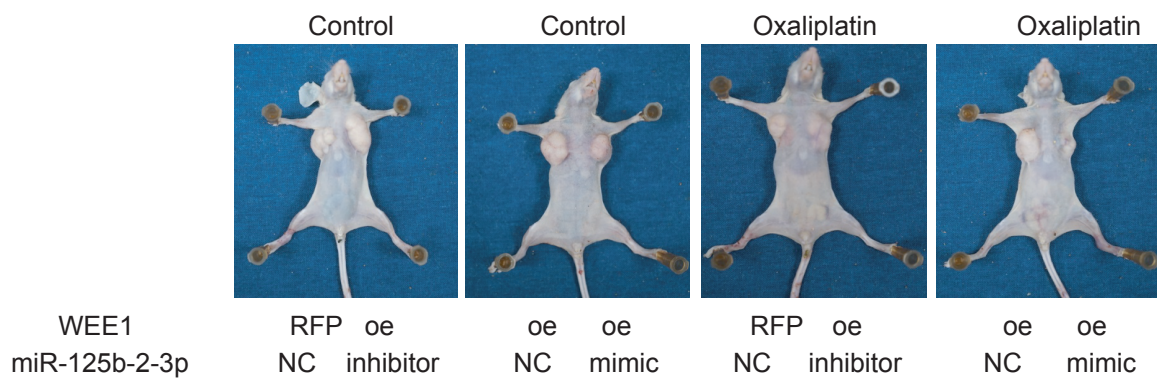

C

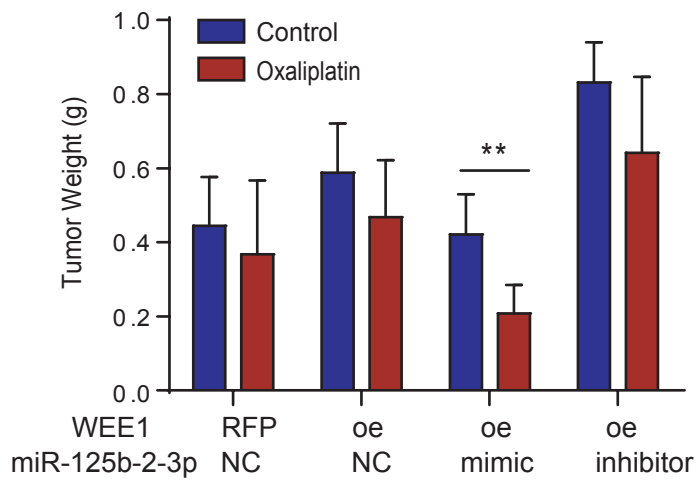

D

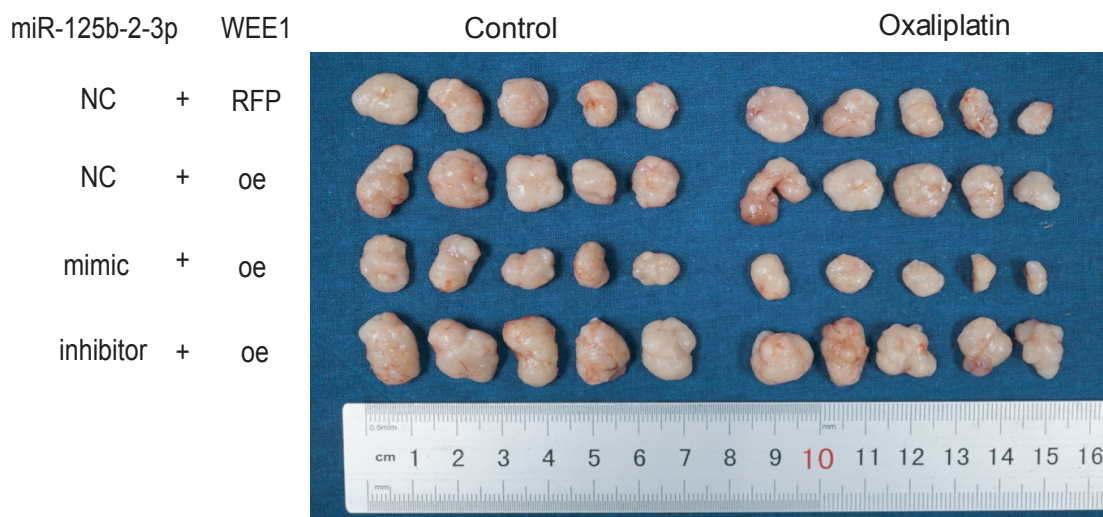

Supplement: Supplementary file 4 — Fig S4 [file CAM4-10-2423-s002.pdf]

A

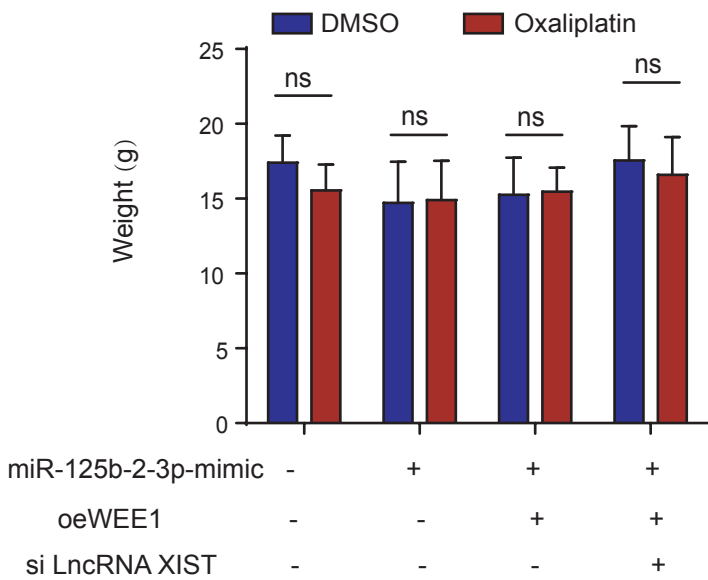

B

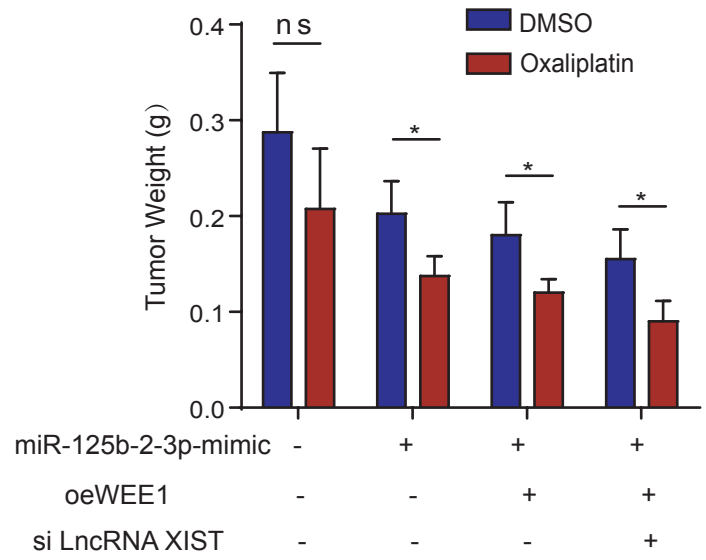

C

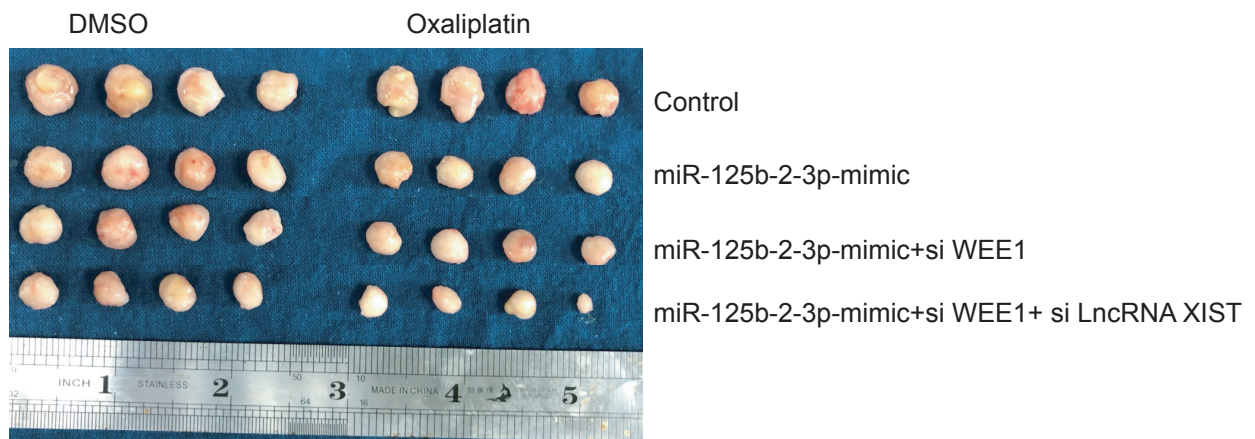

D

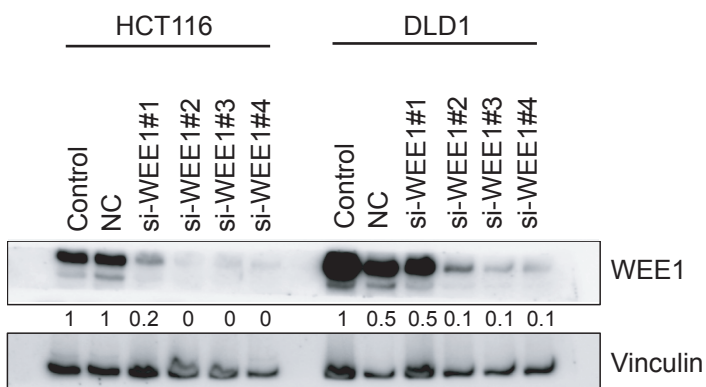

Supplement: Supplementary file 5 — Fig S5 [file CAM4-10-2423-s008.pdf]
