# Supplementary material for: The lncRNA XIST/miR‐125b‐2‐3p axis modulates cell proliferation and chemotherapeutic sensitivity via targeting Wee1 in colorectal cancer
Source: Cancer Med. 2021 Mar 5;10(7):2423–41. doi: 10.1002/cam4.3777 (PMC7982616; doi:10.1002/cam4.3777)
Supplement: Supplementary file 2 — Fig S2 [file CAM4-10-2423-s007.pdf]

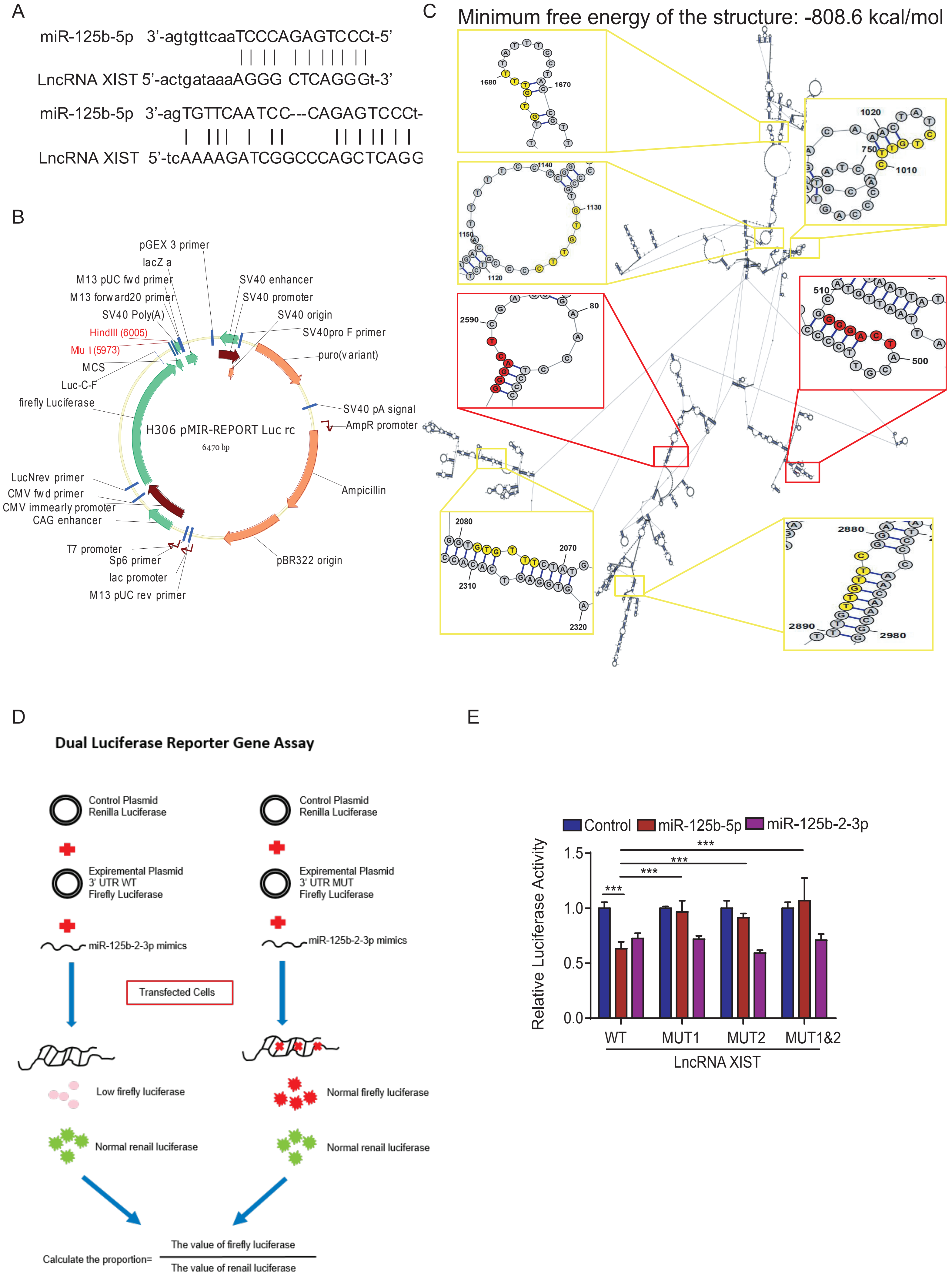
A
miR-125b-5p 3'-agtgttcaaTCCCAGAGTCCCt-5'
LncRNA XIST 5'-actgataaaAGGG CTCAGGGt-3'
miR-125b-5p 3'-agTGTTCAATCC---CAGAGTCCCt-
LncRNA XIST 5'-tcAAAAGATCGGCCCAGCTCAGG
B
pGEX 3 primer
lacZ a
M13 pUC fwd primer
M13 forward20 primer
SV40 Poly(A)
HindIII (6005)
Mlu I (5973)
MCS
Luc-C-F
firefly Luciferase
SV40 enhancer
SV40 promoter
SV40 origin
SV40pro F primer
puro(variant)
SV40 pA signal
AmpR promoter
H306 pMIR-REPORT Luc rc
6470 bp
Ampicillin
pBR322 origin
LucNrev primer
CMV fwd primer
CMV immearly promoter
CAG enhancer
T7 promoter
Sp6 primer
lac promoter
M13 pUC rev primer
C
Minimum free energy of the structure: -808.6 kcal/mol
D
Dual Luciferase Reporter Gene Assay
Control Plasmid
Renilla Luciferase
Expiremental Plasmid
3' UTR WT
Firefly Luciferase
miR-125b-2-3p mimics
Control Plasmid
Renilla Luciferase
Expiremental Plasmid
3' UTR MUT
Firefly Luciferase
miR-125b-2-3p mimics
Transfected Cells
Low firefly luciferase
Normal renail luciferase
Normal firefly luciferase
Normal renail luciferase
Calculate the proportion= The value of firefly luciferase / The value of renail luciferase
E
Control
miR-125b-5p
miR-125b-2-3p
Relative Luciferase Activity
1.5
1.0
0.5
0.0
***
WT
MUT1
MUT2
MUT1&2
LncRNA XIST
