# Supplementary material for: The lncRNA XIST/miR‐125b‐2‐3p axis modulates cell proliferation and chemotherapeutic sensitivity via targeting Wee1 in colorectal cancer
Source: Cancer Med. 2021 Mar 5;10(7):2423–41. doi: 10.1002/cam4.3777 (PMC7982616; doi:10.1002/cam4.3777)
Supplement: Supplementary file 6 — Table S1 [file CAM4-10-2423-s001.docx]

**Table S1:** **The sequences of short-interfere RNA were used in the manuscript.**

| Mimic control | sense  antisense | UUCUCCGAACGUGUCACGUTT  ACGUGACACGUUCGGAGAATT |
| --- | --- | --- |
| Hsa-miR-125b-2-3p mimics | sense  antisense | UCACAAGUCAGGCUCUUGGGAC  CCCAAGAGCCUGACUUGUGAUU |
| MicroRNA Inhibitor NC |  | CAGUACUUUUGUGUAGUACAA |
| Has-miR-125b-2-3p inhibitors |  | GUCCCAAGAGCCUGACUUGUGA |
| Negative control | sense  antisense | UUCUCCGAACGUGUCACGUTT  ACGUGACACGUUCGGAGAATT |
| XIST-Homo-3639 | sense  antisense | GCUGACUACCUGAGAUUUA-TT  UGACUACCUGAGAUUUA-TT |
| XIST-Homo-6167 | sense  antisense | GCAUGCAUCUUGGACAUUUTT  AAAUGUCCAAGAUGCAUGCTT |
| XIST-Homo-7746 | sense  antisense | CCUGUUAACGUAUGUGAUU-TT  AAUCACAUACGUUAACAGG-TT |
| XIST-Homo-8714 | sense  antisense | GCAGUUGUCCACAACCCUATT  UAGGGUUGUGGACAACUGCTT |
| GAPDH Positive control | sense  antisense | UGACCUCAACUACAUGGUUTT  AACCAUGUAGUUGAGGUCATT |
| WEE1-homo-1127 | sense  antisense | GUCCCGGUAUACAACAGAATT  UUCUGUUGUAUACCGGGACTT |
| WEE1-homo-1404 | sense  antisense | GCUGAUGCUAUAAGUGAAATT  UUUCACUUAUAGCAUCAGCTT |
| WEE1-homo-1644 | sense  antisense | GGGCAUGUAACAAGGAUCUTT  AGAUCCUUGUUACAUGCCCTT |
| WEE1-homo-1934 | sense  antisense | GGCACUGGUAAAGCAUUCATT  UGAAUGCUUUACCAGUGCCTT |
| si-h-XIST-7746  (2’ OM3+5’ Chol) | sense  antisense | 5-CCUGUUAACGUAUGUGAUU dTdT-3  5-AAUCACAUACGUUAACAGG dTdT-3 |
| Si-homo-WEE1-1934  (2’ Ome+5’ Chol) | sense  antisense | 5-GGCACUGGUAAAGCAUUCA dTdT-3  5-UGAAUGCUUUACCAGUGCC dTdT-3 |
