# Supplementary material for: The lncRNA XIST/miR‐125b‐2‐3p axis modulates cell proliferation and chemotherapeutic sensitivity via targeting Wee1 in colorectal cancer
Source: Cancer Med. 2021 Mar 5;10(7):2423–41. doi: 10.1002/cam4.3777 (PMC7982616; doi:10.1002/cam4.3777)
Supplement: Supplementary file 7 — Table S2 [file CAM4-10-2423-s003.docx]

| Oxaliplatin | | miR-125b-2-3p expression | | |
| --- | --- | --- | --- | --- |
|  |  | High | Low | Total |
| Response | CR+PR+SD | 30 | 26 | 56 |
|  | PD | 4 | 13 | 17 |
| *P*=0.03 | Total | 34 | 39 | 73 |

Table. S2
