# Supplementary material for: The lncRNA XIST/miR‐125b‐2‐3p axis modulates cell proliferation and chemotherapeutic sensitivity via targeting Wee1 in colorectal cancer
Source: Cancer Med. 2021 Mar 5;10(7):2423–41. doi: 10.1002/cam4.3777 (PMC7982616; doi:10.1002/cam4.3777)
Supplement: Supplementary file 8 — Supplementary Material [file CAM4-10-2423-s006.docx]

**Supplementary**

**Supplementary: Table S1.** The sequences of short-interfere RNA were used in the manuscript.

**Supplementary: Table S2.** The percentages of CRC tissues with high and low miR-125b-2-3p levels were related to the DCRs.

**Supplementary: Figure S1, related to Figure 2.** High expression of miR-125b-2-3p inhibited growth and metastasis, and low expression promoted growth and metastasis. (A) The mimic group increased the volume of miR-125b-2-3p, and the inhibitor group decreased the volume of miR-125b-2-3p in HCT116 cells and DLD1 cells. Error bars, S.D. of three independent experiments. **P* < 0.05 or ***P* < 0.01 versus the corresponding control. (B) Representative images of miR-125b-2-3p expression in CRC tissue chips. Scale bars: 100 μm.

**Supplementary: Figure S2, related to Figure 3.** LncRNA XIST as a competing RNA mediates the function of miR-125b-2-3p. (A) Schematic representation of the predicted 2 target sites for miR-125b-5p in lncRNA XIST. (B) Plasmid construction for luciferase assay. (~~B~~C) The second structure of lncRNA XIST includes the predicted targets of miR-125b-5p (Red) and miR-125b-2-3p (Yellow). Minimum free energy of the structure = -808.6 kcal/mol. (D) Workflow of dual-luciferase reporter gene assay. (E) Luciferase reporter assay in HCT116 cells cotransfected with the reporter plasmid (or the corresponding mutant reporter) and the indicated miRNA mimic. MiR-125b-5p significantly decreased the luciferase activity in XIST-WT but not in XIST-Mut1, XIST-Mut2 and XIST-Mut1 & Mut2. MiR-125b-2-3p decreased the luciferase activity in XIST-WT, XIST-Mut1, XIST-Mut2 and XIST-Mut1&Mut2. Data are presented as the mean ± S.D.. ^*^*P* <0.05 or ^**^*P* <0.01 versus the control.

**Supplementary: Figure S3, related to Figure 4.** MiR-125b-2-3p exerts its function by influencing the target gene WEE1. (A) The expression of WEE1 in CRC cells compared with that in normal tissues, the data from the Gene Expression Profiling Interactive Analysis (GEPIA) database. (B) Schematic representation of the predicted target sites for miR-125b-2-3p in 3’ UTR of WEE1. Data are presented as the mean ± S.D.. ^*^*P* <0.05 or ^**^*P* <0.01 versus the control.

**Supplementary: Figure S4, related to Figure 5.** *In vivo*, miR-125b-2-3p targets WEE1 to influence the function of colorectal cancer cells. (A) HCT116 cells were transduced with RFP and WEE1 lentivirus, and then 2×10^6^ cells were subcutaneously injected into the right and left flanks of nude mice (n=5). The agomir or antagomir were injected into the tumor after the tumor volume reached approximately 100 cm^3^ and then the mice were treated with or without oxaliplatin every two days. Tumor volumes were measured on the indicated days. (B) In the CDX model, the tumor sizes were measured and recorded for each group throughout the experiment (n=5). (B) the representative pictures of mice in each group. (C) The tumor weights were measured and recorded for each group throughout the experiment (n=5). (D) The weights of excised tumors from the eight groups were recorded (n=5). Data are presented as the mean ± S.D.. ^*^*P* <0.05 or ^**^*P* <0.01 versus the control.

**Supplementary: Figure S5, related to Figure 6.** Competing lncRNA XIST mediates the function of miR-125b-2-3p by targeting WEE1. (A) In the PDX models, the mice weights were measured and recorded for each group throughout the experiment (n=5). (B) In the PDX models, the weights of excised tumors from the eight groups were recorded. (C) In the PDX models, the tumor weights were measured and recorded for each group throughout the experiment (n=5). (D) WEE1 protein levels in HCT116 cells and DLD1 cells following the ectopic expression of WEE1 interference. Data are presented as the mean ± S.D.. ^*^*P* <0.05 or ^**^*P* <0.01 versus the control.
